# Utilisation of medical services in Germany and Europe – Results of the European Health Interview Survey (EHIS 3, 2018 – 2020)

**DOI:** 10.25646/12921

**Published:** 2024-11-27

**Authors:** Laura Krause, Franziska Prütz, Anne Starker, Yong Du, Giselle Sarganas, Ronny Kuhnert, Julia Thom, Alexander Rommel

**Affiliations:** Robert Koch Institute, Department of Epidemiology and Health Monitoring, Berlin, Germany

**Keywords:** Outpatient care, Hospital, Dentistry, Psychiatry, Psychotherapy, Early detection of cancer, Medication, Utilisation, Demographics, European Health Interview Survey

## Abstract

**Background:**

Needs-based care is a central concern of healthcare policy. A European comparison of the utilisation of medical services can help to assess national data and identify the need for action.

**Methods:**

This article describes indicators on the utilisation of outpatient and inpatient services, medical examinations and the use of medicines from the third wave of the European Health Interview Survey (EHIS 3) and compares the results from Germany with the European averages.

**Results:**

Age-standardised, the utilisation of medical services in Germany was above the European average without exception, whereby demographic and social differences were similar. Almost all services were utilised more frequently by women and in older age groups. Exceptions are inpatient services and colonoscopy, where there was no difference between the sexes, and more frequent use of psychiatric and psychotherapeutic services and non-prescription medication among younger adults. Specialist medical and dental services were used more frequently in the high education group, psychiatric and psychotherapeutic services as well as inpatient services and prescription medication in the low education group.

**Conclusions:**

The European comparison requires a differentiated categorisation of the findings. While the high utilisation in Germany for some evidence-based services (e.g. colonoscopy, dentistry) indicates good access to care, the high inpatient utilisation, for example, is also the subject of critical discussion.

## 1. Introduction

Healthcare and prevention are important tasks of the healthcare system and are essential for maintaining and improving the health of the population [1]. Ensuring access to needs-orientated and evidence-based medical care is a central concern of national and European health policy. European countries and their different healthcare systems face similar challenges. These include, above all, demographic change and the associated increase in non-communicable chronic diseases such as diabetes, cardiovascular diseases and cancer [2]. Against this background, it is important to compare indicators of healthcare provision and the utilisation of medical services in a European context in order to assess the national data and, if necessary, derive the need for action.

A general distinction is made in healthcare between the outpatient and inpatient sectors. In Germany, outpatient care is mainly provided by registered doctors, psychotherapists and dentists. They are usually the first place to go in the professional medical care system. In the case of health complaints, they determine the need for treatment, carry out examinations and treatments and, if necessary, arrange for further health and social services [3]. Around 90 % of adults in Germany receive outpatient medical or psychotherapeutic services every year [4], and just over 80 % receive dental examinations and treatment [5].

Inpatient hospital care is utilised by around 16 % of the population in Germany within a year; utilisation is strongly age-dependent [6]. Compared to other European countries, Germany has one of the highest densities of hospital beds [7].

Medical care also includes check-ups for blood pressure, cholesterol and blood sugar. These play an important role in the prevention, diagnosis and management of diabetes and cardiovascular disease and are important aspects of the quality of care. High blood pressure, hyperlipidaemia and diabetes are generally regarded as chronic diseases and important modifiable risk factors for cardiovascular disease. However, they remain unrecognised in the early stages because they show no symptoms. The last national survey showed that in Germany almost one in five adults with high blood pressure [8], more than half of those affected by hyperlipidaemia [9] and almost a quarter of all people with diabetes [10] had not yet received a diagnosis at the time of the survey. Meanwhile, undiagnosed hypertension [11] and undiagnosed diabetes [12] have decreased in the adult population, and blood pressure check-ups have increased significantly among people with known hypertension [13].

The supply of medication also plays an important role in the treatment of health impairments, disorders and illnesses. In Germany, more than half of the population takes medication prescribed by a doctor within two weeks [14].

Preventive healthcare services include vaccinations or cancer screening, for example. Since July 2019, colorectal cancer screening has been offered in Germany as an organised programme with an invitation and information system for the early detection of colorectal cancer. Until then, according to data from the statutory health insurance around 15 % of those eligible had a colonoscopy (2009 – 2018) [15]. In addition to the early detection of colorectal cancer, colonoscopy is also used to clarify symptoms, so that the utilisation of this examination is higher overall [16].

This article contains key data on the utilisation of medical care among adults in Germany and Europe from the third wave of the European Health Interview Survey (EHIS 3), which was conducted between 2018 and 2020 [17]. Results on the utilisation of general and specialist medical care, dental care, psychiatric and psychotherapeutic care, hospital care and selected outpatient services - colonoscopy, monitoring of blood pressure, blood lipids and blood sugar by healthcare professionals and the use of medication - are presented. Gender, age and education are examined as important factors influencing the utilisation of medical care [18–20] and differences between Germany and the European average are shown.

**Key statements**

► The age-standardised utilisation of healthcare services in Germany is above the European average.
► Demographic and social differences are similar in Germany and Europe.
► With few exceptions, healthcare services are used more frequently by women and in higher age groups.
► Specialist and dental services are more frequently used in the high education group, while psychiatric/psychotherapeutic and inpatient services and medicines are more frequently used in the low education group.
► The findings for Germany need to be interpreted in a differentiated way: the high use of evidence-based services indicates good access to care, while the high use of inpatient care is also discussed critically.

## 2. Methods

### 2.1 Sample design and study conduct

EHIS 3 was legally binding in all EU member states. The basis for this is the European Commission Regulation (EU) 2018/255 of 19 February 2018 implementing Regulation (EC) No 1338/2008 of the European Parliament and of the Council on Community statistics on public health and health and safety at work with respect to statistics based on the EHIS [21]. The aim of the EHIS survey is to regularly provide comparable health data from the EU Member States and thus enable the development of health indicators in Europe to be analysed. The target population is the population aged 15 years or older living in private households and residing in the country’s territory. EHIS 3 was conducted in 2019 in all EU Member States as well as Iceland, Norway, Albania, Serbia and Turkey. Some countries were granted an exemption regarding the data collection period, so that data is available from 2018 to 2020. A quality report contains detailed information on the methodological approach of the individual countries [22]. The aggregated data can be found on the website of the Statistical Office of the European Union [23]. Albania, France, Turkey and the United Kingdom have not yet made any data publicly available [24]. For research purposes, anonymised data at participant level (microdata) for the EU Member States can be requested from Eurostat [25]. The dataset used for the present analyses contains data from 29 European countries (Austria, Belgium, Bulgaria, Croatia, Cyprus, Czech Republic, Denmark, Estonia, Finland, Germany, Greece, Hungary, Iceland, Ireland, Italy, Latvia, Lithuania, Luxembourg, Malta, Netherlands, Norway, Poland, Portugal, Romania, Serbia, Slovakia, Slovenia, Spain and Sweden). In the national EHIS implementation, countries can choose different types of data collection and combinations thereof. Personal interviews and telephone surveys are used. In addition, self-completion questionnaires are used for data collection by post and on the Internet [22].

### 2.2 Indicators

The regulation on the implementation of the EHIS specified the items to be surveyed, including their response categories and the codes to be transmitted to Eurostat. In addition, the wording of the questions and their response categories as well as the order in which they are asked were explained in a methodological manual and made available in the form of a sample questionnaire [17]. Compliance with the rules and recommendations designed as guidelines was essential to ensure harmonised and high-quality health data in the EU.

#### Utilisation of outpatient medical services

The utilisation of general practitioners (GP) was measured with the question: ‘When did you last consult a general practitioner or family doctor for advice, examination or treatment?’ The term ‘family doctor’ includes services provided by internists working as family doctors. Respondents could choose between the answers ‘Less than 12 months ago’, ‘12 months ago or longer’ and ‘Never’. The same wording was used to ask about visits to other specialists. Two dichotomous variables were formed to distinguish respondents who had consulted a family doctor or general practitioner (hereafter referred to as ‘consultation with a general practitioner’ or ‘GP’) and those who had consulted a specialist in the last twelve months from respondents who had not done so.

#### Utilisation of dental services

Participants were asked: ‘When was the last time you visited a dentist or orthodontist on your own behalf (that is, not while only accompanying a child, spouse, etc.)? Would you say – ‘Less than 6 months’, ‘6 to less than 12 months’, ‘12 months or longer’ or ‘Never’.’ The first and the last two categories were combined for the analyses. In this way, the indicator of the 12-month prevalence of dental utilization (yes/no) is obtained [5].

#### Utilisation of psychiatric and psychotherapeutic services

For mental health complaints and disorders, the utilization of specialized care was ascertained specifically. The participants were asked: ‘In the past 12 months, have you seen a psychologist, psychotherapist, or psychiatrist for consultation, examination, or treatment?’ Response options were ‘Yes,’ ‘No,’ ‘Don’t know,’ and ‘No answer’. In the following, the term ‘psychotherapeutic and psychiatric’ services is used in summary, whereby services provided by psychologists without a license, for example in the context of outpatient addiction counselling, are also included.

#### Utilisation of inpatient medical services

Hospital stays were measured using the question: ‘Have you, as an inpatient, spent one night or more in hospital in the last 12 months? This does not include stays in accident and emergency departments or as an outpatient without an overnight stay.’ The question could be answered, Yes’ or ‘No’.

#### Utilisation of colonoscopy

Utilisation of colonoscopy was assessed with the question ‘When was the last time you had a colonoscopy?’ The following answer options were available: ‘Within the past 12 months’, ‘Between 1 and less than 5 years ago’, ‘Between 5 and less than 10 years ago’, ‘10 years ago or more’ and ‘Never’. Over the past two decades, comprehensive colorectal cancer screening programmes have been implemented in European countries, varying by time of introduction, programme type, screening test used, and target age group [26]. For the analyses, the utilisation of the last colonoscopy within the last ten years is presented for women and men aged 50 to 74 years, as this population is the target group for the recommendation to introduce organised screening for the early detection of colon cancer at this interval [27], which has already been implemented by many countries.

#### Utilisation of medical examinations

Data on blood pressure measurement by healthcare professionals were collected using the question ‘When was the last time you had your blood pressure measured by a healthcare professional?’ Five response options were given for this question: ‘Within the last 12 months’, ‘Between 1 and less than 3 years ago’, ‘Between 3 and less than 5 years ago’, ‘5 years or more ago’ and ‘Never’. The response should refer to blood pressure measured by a healthcare professional and not by the respondent or a family member/relative.

Using analogous wording, data on the measurement of blood lipids and blood sugar by healthcare professionals in the last twelve months were collected. Based on the responses, a dichotomous variable for the measurement of blood pressure, blood lipids and blood sugar in the last twelve months was created (yes/no).

#### Use of medication

The use of medication in the last two weeks prior to the survey reflects the prevalence of current use. A distinction is made between the use of medication prescribed by a doctor and the use of freely available preparations, and the following questions are used: 1. ‘Have you taken medication prescribed by a doctor in the last two weeks? This does not include the contraceptive pill or other hormonal contraceptives.’ 2. ‘In the last 2 weeks, have you taken any medication, herbal remedies or vitamins that were not prescribed by a doctor? This does not include the contraceptive pill or other hormonal contraceptives.’ Response options were ‘Yes,’ ‘No,’ ‘Don’t know,’ and ‘No answer’.

#### Sociodemographics

In addition to the sex of the respondents, age (in categories) was also considered as a determinant of utilisation. The following age group categorisation was used for most indicators: 18 – 29 years, 30 – 44 years, 45 – 64 years and 65 years and older. For the utilisation of dental services, the oldest age group was divided into two groups, 65 – 74 years and 75 years and older. This categorisation is recommended by the World Health Organization (WHO) for oral health [28]. Colonoscopy utilisation is reported from the age of 50 years in 5-year age groups (50 – 54 years, 55 – 59 years, 60 – 64 years, 65 – 70 years and 70 – 74 years). Education was also examined as a determinant of utilisation. The International Standard Classification of Education (ISCED) was used to categorise the respondents’ information on education [29]. ISCED considers both school and vocational educational qualifications and is particularly suitable for international comparisons. For the analyses, the ISCED categories 0 to 2 were combined into a low, 3 to 4 into a medium and 5 to 8 into a high education group.

### 2.3 Statistical methods

The analyses are based on data from a total of 256,202 participants (136,882 women, 119,320 men) aged 18 and over who answered the EHIS survey themselves. For Malta and Iceland, data is available from the age of 20. Table 1 shows the case numbers for all utilisation indicators.

The analyses were calculated using a weighting factor in order to take each country into account in proportion to its population size. In order to compensate for potentially distorting age differences between the countries, a direct age standardisation was carried out. The age structures of the country samples were adjusted to the European standard population for 2013 [30]. For each of the indicators analysed, the prevalence was calculated stratified by gender, age and education with 95 % confidence intervals (95 % CI). A statistically significant difference between groups is assumed if the corresponding p-value is less than 0.05. Differences by age, gender or education are only discussed in the text if they are statistically significant. The group differences were calculated using a chi-square test (adjusted according to Rao & Scott). The survey methods were used for this purpose. The household ID was considered as a cluster. All analyses were carried out using the program R version R 4.4.1 (packages: tidyverse 2.0.0, srvyr 1.3.0, readstata 13 0.10.1) and STATA version 17.0. Due to different statistical methods, there may be deviations in detail from previously published results on the utilisation of medical services for Germany.

## 3. Results

### 3.1 Utilisation of outpatient medical services

**Figure 1:**
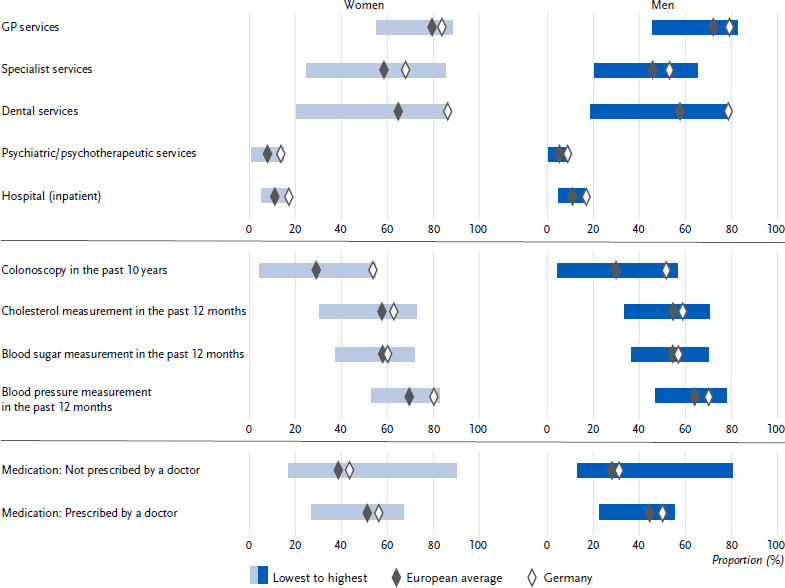
Age-standardised prevalence of the utilisation of medical services by gender for Germany and Europe. Source: EHIS wave 3 (2018 – 2020)

**Figure 2:**
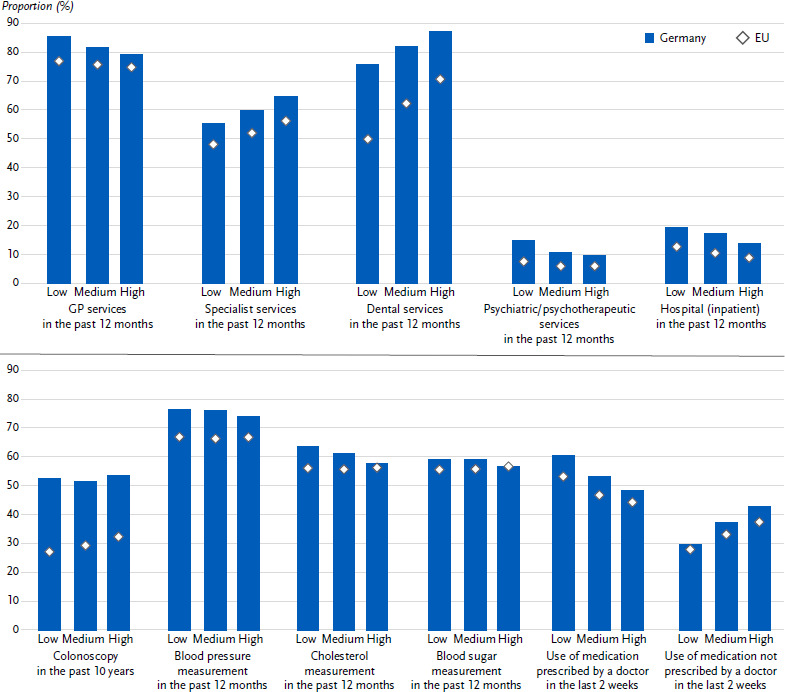
Age-standardised prevalence of utilisation of medical services by education for Germany and Europe. Source: EHIS wave 3 (2018 – 2020)

Overall, 81.4 % of respondents in Germany had used general practitioner services in the year prior to the survey. This applied significantly more often to women (83.6 %) than to men (79.1 %; Figure 1). The 12-month prevalence of GP service use increased significantly across age groups in Germany, from 77.7 % among 18- to 29-year-olds to 88.1 % among those aged 65 and over (Annex Table 1). Furthermore, in Germany, a significantly higher 12-month prevalence of utilisation of GP services was found among persons in the low education group compared to persons in the high education group (Figure 2). Compared to Germany, the utilisation of GP services in Europe was 75.7 % on average and thus about 6 percentage points lower, with a similarly higher utilisation among women and a similar age range (Figure 1, Annex Table 1). On average, educational differences are only weakly pronounced on average on the European level, with around three quarters of people having used GP services in the last year, regardless of their education group (Figure 2).

Specialist services were utilised by 60.4 % of the respondents in Germany in the year prior to the survey. As with the utilisation of general practitioners, a significantly higher utilisation was found among women compared to men (67.8 % and 53.0 % respectively; Figure 1). The 12-month prevalence of specialist care utilisation increased significantly with age, from 52.6 % among 18-to 29-year-olds to 66.1 % among those aged 65 and over (Annex Table 1). In both Germany and on average in Europe, there was a significant educational gradient in the 12-month prevalence of specialist care (Figure 2). Overall, the utilisation of specialist services in Europe (52.1 %) is significantly lower than in Germany. Women also utilised specialist services more often than men in the last 12 months, and the age pattern was similar, although the difference between the youngest and oldest age groups was greater than in Germany (Annex Table 1).

### 3.2 Utilisation of dental services

Overall, 82.3 % of respondents in Germany had utilised dental services in the year prior to the survey, women (86.1 %) significantly more often than men (78.6 %) (Figure 1). The 12-month prevalence of dental care utilisation in Germany was over 80 % in almost all age groups; it was only significantly lower among the very old aged 75 and over at 77.4 % (Annex Table 2). The results also indicate an educational gradient in the 12-month prevalence of dental care utilisation in Germany (Figure 2): Utilisation was significantly lower among people in the low education group (75.6 %) than among people in the high education group (87.0 %). Compared to Germany, the European average utilisation of dental services was significantly lower by more than 20 percentage points (61.1 %). The observed sex difference in favour of women was reflected at a lower level in the European average (Figure 1). The age progression was similar in the European average, but a significant decline in the utilisation of dental services was already observed among 65- to 74-year-olds (Annex Table 2). An educational gradient was also observed in the European average, which was more pronounced than in Germany, with a difference of 20 percentage points between the low and the high education group (Figure 2).

### 3.3 Utilisation of psychiatric and psychotherapeutic services

The proportion of people in Germany who accessed psychotherapeutic and psychiatric services within a year was 11.1 %, with a significant difference between women (13.5 %) and men (8.7 %) (Figure 1). At 14.4 %, the use of psychiatric and psychotherapeutic services was highest among 18- to 29-year-olds. Only 4.8 % of individuals aged 65 and older sought psychiatric and psychotherapeutic help. The age gradient was more pronounced among women than among men (Annex Table 3). With respect to education, it was found that the proportion of individuals in Germany accessing psychiatric and psychotherapeutic care was significantly higher by 5.5 percentage points in the lower education group than in the middle and high education groups. The European average for utilization of psychiatric and psychotherapeutic services was considerably lower at 6.4 %, though there was also a significant gender difference (women: 7.7 %, men: 5.1 %; Figure 1). The age pattern in utilization at the European level was similar, though at a lower level (Annex Table 3). Differences in education were also evident in the European average, but at 1.6 percentage points the difference was smaller than in Germany (Figure 2).

### 3.4 Utilisation of inpatient medical services

Around one sixth (16.9 %) of adults in Germany spent at least one night as an inpatient in a hospital during the year. There were virtually no differences between women (17.0 %) and men (16.8 %) (Figure 1). The proportion of people with at least one inpatient stay per year increased with age: while 11.4 % of 18- to 29-year-olds used inpatient care, the proportion rose to 17.8 % of 45- to 64-year-olds and 26.3 % of those aged 75 and over (significant differences; Annex Table 1). The European average for the use of inpatient care was 10.8 % (women: 10.9 %, men: 10.8 %) and thus significantly lower than in Germany. In Germany, as at European level, there were significant differences in hospital use by education group. The proportion of people in the low education group who used inpatient services was 5.5 percentage points higher in Germany and 3.8 percentage points higher in the European average than in the high education group (Figure 2). The European average also showed an increase with age, although the figures were lower than in Germany (18-29 years: 6.7 %, 75 years and older: 20.6 %; Annex Table 1).

### 3.5 Utilisation of colonoscopy

The percentage of persons who stated that they had had a colonoscopy in the last 10 years was 52.6 % in Germany, with hardly any differences between women (53.6 %) and men (51.6 %). The percentage of persons who have had a colonoscopy increased significantly with age in both women and men, from 36.7 % among 50- to 54-year-olds to 66.8 % among 70- to 74-year-olds (Annex Table 3). Overall, Germany was clearly above the European average of 29.3 % in the use of colonoscopies (Figure 1). Regarding education, there were no differences in Germany, whereas the European average showed a significant gradient to the disadvantage of the lower education group (Figure 2). However, the difference between the lower education group (27.2 %) and the higher education group (32.4 %) is relatively small at 5 percentage points.

### 3.6 Blood pressure, blood lipids and blood sugar measured by healthcare professionals

The percentage of individuals who had their blood pressure, blood lipids and blood sugar measured by healthcare professionals in the last twelve months was 75.1 %, 60.7 % and 58.5 %, respectively. The percentage for women was significantly higher than for men (Figure 1). The proportion of people having their blood pressure, blood lipids and blood sugar measured rose significantly with age, reaching 87.3 %, 80.2 % and 78.4 %, respectively, among those aged 65 and over (Annex Table 1). In terms of educational attainment level, no educational gap was found in Germany with regard to the measurement of blood pressure and blood sugar (Figure 2). However, people in the high education group had a lower proportion of blood lipid measurement (57.4 %) than people with low (63.1 %) and medium (60.9 %) education. In relation to the European population, Germany was 8.4 percentage points, 4.6 percentage points and 2.4 percentage points above the EU average in terms of measurement of blood pressure, blood lipids and blood sugar levels, respectively (Figure 1). Standardised to the Europe population, Germany was similar to the European average in terms of measurement of blood pressure, blood lipids and blood sugar levels (Annex Table 1). On average in Europe, no educational differences were found in the measurement of blood pressure, blood lipids and blood sugar (Figure 2).

### 3.7 Use of medication

More than half of people in Germany (53.1 %) reported having taken medication prescribed by a doctor in the last two weeks. The proportion was higher for women (56.2 %) than for men (50.0 %) (Figure 1). In Germany, the prevalence of medication use differed significantly between life stages and increased with age: the use prevalence among 65-year-olds and older was much higher than in younger age groups (Annex Table 1). In Germany, however, the prevalence use of women and men equalised from the age group of 65 years and older (Annex Table 1). Compared to Germany, the European average of prevalence use was lower (47.8 %). Gender differences in the use of prescribed medication were found in all age groups of the European average, with significantly higher prevalence of use among women than men (Annex Table 1). Also, in the European average, prevalence use differed significantly between life stages and increased with age (Annex Table 1). Both in Germany and in the European average, people in the low education group had significantly higher prevalence rates in the use of prescribed medication than people in the high education group (Figure 2).

Overall, 37.3 % of people in Germany reported that they had taken medication not prescribed by a doctor in the last two weeks. Women (43.5 %) were significantly more likely to report this than men (31.1 %) (Figure 1). The prevalence of taking medication not prescribed by a doctor tended to decrease with age: In Germany, 39.2 % of 18 to 29-year-olds had taken medication not prescribed by a doctor in the last two weeks, while the prevalence use among 65-year-olds and older was 31.2 % (Annex Table 1). Compared to Germany, the use of non-prescribed medication was lower than in the European average (33.3 %), and there were also gender differences in the use of non-prescribed medication, with significantly higher prevalence of use among women of all age groups (Figure 1 Annex Table 1). The age trend also showed a slight downward trend in the European average (Annex Table 1). Both in Germany and in the European average, people in the high education group showed significantly higher prevalences in the use of medication not prescribed by a doctor (Figure 2).

## 4. Discussion

The main finding was that, without exception, the utilisation of the medical services examined was higher in Germany than the European average. However, demographic and social differences were similar in Germany and Europe. For most services, utilisation was higher among women and with increasing age. Social gradients did not follow a uniform pattern. Services whose utilisation did not differ according to education (e.g. colonoscopy) were also found, as well as a higher utilisation with lower education (e.g. inpatient utilisation) as well as with higher education (e.g. specialist and dental services).

### 4.1 Utilisation of outpatient medical services

The utilisation of outpatient medical services in Germany was at a relatively high level compared to the European average. Both general practitioner and specialist services were used significantly more often on average than in other European countries. Furthermore, similar patterns were found in Germany and Europe in regard of an increase in utilisation with age and more frequent use of outpatient medical services by women. In terms of education, the use of specialist services increased with higher education, but the use of general practitioners decreased.

While the age-associated increase in utilisation can be explained by greater morbidity in old age, various explanatory approaches can be used for gender differences. On the one hand, women seek professional help more quickly when they face health problems. On the other hand, women-specific gynaecological examinations or cancer screenings can influence utilisation, and women generally take advantage of preventive services more regularly than men [31].

There are various reasons for the higher level of utilisation in Germany compared to most other European countries. In Germany, free access to outpatient doctors can be emphasised as a specific factor in the utilisation of outpatient medical services. The German healthcare system has hardly any distinct gatekeeper function that could regulate the use of specialist medical services. The only exceptions are the voluntary programmes of GP-centred care, which are only used by a small proportion of the population and service providers [32]. In addition, the German healthcare system has a comparatively very high level of coverage of health services in various areas [33]. This is accompanied by a relatively low level of out-of-pocket payments and unmet care needs [33].

The differences shown according to education, in particular in favour of higher utilisation of specialist services, are known from the literature. They are explained, among other things, by a greater tendency among people in the lower education group to be more willing to be ‘guided’ through the care system by their GPs, partly due to communication barriers, whereas many members of higher education groups seek direct access to specialists [18, 34]. Furthermore, the higher morbidity in low socioeconomic status groups should be considered [35]. The extent to which the education-specific mix of general and specialist medical care is suitable for ensuring needs-based care in the case of different morbidity should be the subject of further research in Germany and beyond. In doing so, it is also important to consider potential structural access barriers, such as longer distances and waiting times for those with statutory health insurance, which can influence utilisation behaviour.

### 4.2 Utilisation of dental services

The majority of adults in Germany (82.3 %) have utilised dental services within one year. Compared to EHIS 2, which was conducted in 2014/2015, utilisation has thus remained stable at a high level (81.6 %) [5]. The European average utilisation rate was more than 20 percentage points lower (61.1 %). Dentistry is a discipline that also has a preventive focus, particularly in Germany [36]. Here, people with statutory health insurance aged 18 and over are entitled to a dental check-up once every six months [37]. Utilisation is documented once a year in a bonus booklet in order to be entitled to a higher fixed subsidy for dentures if necessary [36]. In this respect, the higher utilisation of dental services in Germany compared to the European average should also be seen in the context of the bonus system for dentures [36].

In Germany, a higher utilisation rate of dental services was found among women, people of middle and older age and in the high education group. This is supported by earlier research [5, 38]. Overall, this is consistent with the results of studies showing that these groups of people are more prevention-orientated [39, 40]. These differences by sex, age and education were also found in the European average. Despite the preventive nature of dentistry, treatment still predominates in dental care for adults in Germany [36, 41]. The decline in preventive dental check-ups during the COVID-19 pandemic [42–44] further favours therapeutic-curative dentistry. In an international comparison, contract dental care in Germany offers those with statutory health insurance a wide range of services. For example, services such as fixed dentures are not covered by health insurance in many other countries [36], while in Germany the basic treatment for dentures is covered by statutory health insurance if the patient has kept a complete bonus booklet for more than ten years (standard care) [45]. The National Association of Statutory Health Insurance Dentists (Kassenzahnärztliche Bundesvereinigung, KZBV) rates the overall availability of contracted dental care in Germany as good, despite regional variations and an urban-rural gap [44].

### 4.3 Utilisation of psychiatric and psychotherapeutic services

With a rate of 13.5 % among women and 8.7 % among men, the utilization of psychotherapeutic and psychiatric services in Germany has remained largely stable from EHIS 2 to EHIS 3 (women: 12.8 %, men: 8.9 % [46]). The fact that individuals aged 65 and older report significantly fewer contacts with psychiatrists, psychotherapists, and psychologists than the 18- to 29-year-olds may be related to stigmatising attitudes among older people towards mental disorders and seeking help, as well as negative assumptions about professional services [47]. In contrast, the higher utilization of psychiatric and psychotherapeutic services by women compared to men, and by people with a low level of education than by those with a higher level of education, corresponds to the known gender- and education-related gradients in disease burden [48]. However, as demonstrated for psychotherapeutic care, for example [49], there is still potential for more needs-based access. This also includes the very uneven regional distribution of psychotherapeutic and psychiatric practices, which poses a barrier to the use of these services and is partially reflected in regionally varying utilization patterns [46].

Compared to the European average of 6.4 %, psychiatric and psychotherapeutic services are utilized far more frequently in Germany (11.1 %). This difference is likely explained largely by the coverage of these services through statutory (and often private) health insurance in Germany as well as the broad range of care services available for people with mental complaints and disorders, which allows many adults at least one-time contact with psychologists, psychotherapists, or psychiatrists as measured by the survey [50, 51]. Nevertheless, given the prevalence of mental disorders in the population, the frequency of the use of specialized care might indicate a potential underprovision of professional help in Germany [52]. Assuming rather minor differences in the prevalence of mental disorders across Europe, this underprovision may be even more pronounced in many other EU countries.

### 4.4 Utilisation of inpatient services

At around 17 %, the proportion of adults in Germany who have spent at least one night in hospital as inpatients within a year has risen slightly compared to the EHIS 2 survey (16.2 %) [6]. This may be due, among other things, to the ageing population and the associated higher burden of disease; another reason cited is the increase in treatment options due to advances in medical technology [53]. The finding of higher utilisation of inpatient services among younger women [6] in EHIS 2 is hardly found in the current EHIS data. Whether this indicates a persistent trend, which could possibly be related to a higher proportion of outpatient hospital births, will be shown in follow-up surveys. The higher utilisation of hospital services among older people can be explained by a higher need for treatment due to increased morbidity in old age. Educational differences in inpatient utilisation can also be found in earlier waves of the EHIS and can be attributed to a higher prevalence of chronic diseases, especially in the low education groups [54].

The high level of inpatient utilisation in Germany compared to the rest of Europe – the European average is only just over 10 % – is also reflected in the official hospital statistics. According to these statistics, in 2020 Germany had the second highest number of hospital discharges per 100,000 inhabitants in Europe [7]. The high level of inpatient utilisation is associated, among other things, with a high number of elective procedures such as hip and knee replacements compared to the rest of Europe, as well as a high level of so-called outpatient-sensitive services (inpatient services that could also be provided in the outpatient sector) [55]. This is often seen in the context of healthcare structures: Germany has the highest number of hospital beds per 1,000 inhabitants in Europe (2020: 7.8 compared to 5.0 in the EU23 [7]). However, the higher burden of disease in Germany due to the demographic structure of the population may also contribute to the higher inpatient utilisation. A report by the German Hospital Institute suggests that follow-up care and intersectoral cooperation are better organised in other countries, which reduces the burden on hospitals [56]. Furthermore, the system of lump-sum DRGs used for billing provides financial incentives not only for hospital services that generate higher profits, but also for increasing in the number of cases in general [53]. The fact that, in addition to public and not-for-profit providers, around 40 % of hospitals in Germany are privately owned [57] and therefore profit-driven, may also have an additional effect [58]) Overall, there is a perceived need for political action in the area of hospital care: a government commission was set up in May 2022 with the aim of reforming hospital care [59].

### 4.5 Utilisation of colonoscopy

Since 2002, colonoscopy has been offered in Germany as a cancer screening test (preventive colonoscopy). An organised and quality-assured screening programme has been established since July 2019. Since then, people with statutory health insurance have been informed of this offer and invited to attend. Women aged 50 to 54 can choose to take an annual test for faecal occult blood. Men aged 50 to 54 years can choose between an annual faecal occult blood test and a colonoscopy (every ten years). Women and men aged 55 years and older can choose between a faecal occult blood test, which is performed every two years, and a maximum of two screening colonoscopies at ten-year interval. If the faecal occult blood tests are abnormal, there is always an entitlement to a colonoscopy [60]. However, it is also carried out to investigate symptoms or other diseases (curative colonoscopy). Based on data from the statutory health insurance, it could be shown that the majority of examinations were carried out for reasons other than early detection; the ratio between preventive and curative colonoscopies was about 1:3 [61]. Both examinations are covered by statutory health insurance in Germany, and there is a well-developed range of gastroenterology services [62].

Over the past two decades, comprehensive colorectal cancer screening programmes have been implemented in European countries, but the services they offer differ considerably [26]. However, EHIS 3 only asks about participation, not the reason for the examination, so it can be assumed that both options are reported here. The utilisation of colonoscopies in Germany has remained relatively constant compared to EHIS 2 (2014/2015) [63]. The comparably high proportion of colonoscopies in Germany has also been found in other studies based on self-reports [64]. Already in EHIS 2 there were large differences in utilisation in Europe, with overall findings indicating that uptake was highest in countries with fully implemented screening programmes or in countries offering both faecal tests and colonoscopy for colorectal screening, and much lower or almost non-existent in countries without a screening programme [26]. The youngest age group eligible for screening was also associated with lower uptake in EHIS 2, as was a low level of education [26, 65].

### 4.6 Blood pressure, blood lipids and blood sugar measured by healthcare professionals

The majority of adults in Germany had their blood pressure, blood lipids and blood sugar measured at least once by healthcare professionals in the year prior to the survey. These results are above the European average. In both Germany and other European countries, women had their blood pressure, blood lipids and blood sugar measured more often than men and older adults more often than younger adults. No educational differences were found in the proportions of blood pressure and blood lipid measurement in Germany and in the European average. However, women in the high education group in Germany had their blood lipids measured less often than women in the low and middle education groups.

Germany is one of the European countries with an above-average proportion of people who have had their blood pressure, blood lipids and blood sugar measured in the past 12 months. This was consistently found in persons aged 15 and over in EHIS 2 (2014/2015) [66] and EHIS wave 3 (2018 – 2020) [67]. According to EHIS 2, 77.6 %, 56.1 % and 58.1 % of people over 15 years of age in Germany had their blood pressure, blood lipids and blood sugar measured [66], which is comparable to the results of the present study in adults aged 18 and over. In EHIS 2, age and gender differences in the measurement of blood pressure, blood lipid and blood sugar were also found, both for Germany and for the other European countries [66].

The high proportion of preventive medical check-ups reflects, on the one hand, the health awareness of people without known illnesses (i.e. hypertension, hyperlipidaemia and diabetes) and, on the other hand, the quality of care for people with these known illnesses. People with known illnesses who are undergoing treatment contribute significantly to the high proportions found in this study. Regular measurements of blood pressure, blood lipid and blood sugar for people with these known illnesses are necessary, not only for drug therapy adjusting the dose of medication, but also for monitoring the course of the disease. These measurements are required by clinical guidelines. For example, the DMP guideline for type 2 diabetes (DMP: disease management programme) recommends that blood sugar and blood pressure levels in people with type 2 diabetes should be measured quarterly, but at least every six months [68]. The clinical guidelines also recommend treatment targets for blood pressure, blood lipids and blood sugar for people undergoing therapy [69]. As a result, almost all (95.7 %) people with type 2 diabetes over the age of 45 years have their blood sugar (HbA1c) determined in the past 12 months [70]. Since hypertension [71], hyperlipidaemia [9] and diabetes [72] are highly age-related chronic diseases, it is not unexpected that the proportion of people who have their blood pressure, blood lipids and blood glucose measured increases markedly with age, reaching high levels in adults aged 65 years and older.

For people with undiagnosed hypertension [11], dyslipidaemia [9] and diabetes [12], regular measurement of blood pressure, blood lipids and blood glucose by healthcare professionals can detect these diseases at an earlier stage. In order to increase participation in preventive medical check-ups, all health insurance funds in Germany offer their policyholders so-called bonus programmes. In addition, anyone over the age of 35 with statutory health insurance is entitled to a medical examination every three years, which includes a blood pressure measurement and a blood test to determine blood sugar and cholesterol levels [73]. Starting in April 2019, this health check-up was extended to include 18- to 34-year-olds, but only once [74]. Data from statutory health insurance funds show that about three quarters of all insured persons aged 35 and older have ever taken part in a health checkup [75] and almost half have done so in the last two years [76]. For example, 78.1 % of women and 62.5 % of men without known hypertension had their blood pressure measured by a healthcare professional within the last year [77]. The high rate of participation in preventive medical check-ups and the financial incentives for health care through bonus programmes offered by health insurance companies may have contributed to the high proportion of preventive medical check-ups in Germany.

The education-stratified results show that the measurement of blood lipid levels in adults in Germany varies considerably between the different education groups. An earlier analysis of EHIS 2 for Germany showed that, in particular, women in the high education group had lower rates of blood lipid and blood sugar measurements than women in the medium and low education groups [66]. This could be due to the fact that in Germany, the educational differences in the prevalence of cardiovascular diseases [71, 78] and diabetes [72] are particularly pronounced in women. Women with a higher level of educational attainment have a significantly lower prevalence of cardiovascular diseases and diabetes than women with a lower level of educational attainment. In men, however, these differences were less pronounced. However, the educational gap in blood sugar measurement in women, which was weighted to the general adult population in Germany [19], disappeared in the present analysis when data were weighted to the European standard population.

### 4.7 Use of medication

More than half of adults in Germany took prescribed medication in the two weeks prior to the survey. This prevalence has remained similar compared to EHIS 2 [79]. Significant gender-related differences in the use of medically prescribed drugs were observed in both EHIS 2 and EHIS 3, especially in the younger age groups (under 65 years), with higher prevalence use among women than among men. From the age of 65, the prevalence rates for women and men equalise. The use of prescribed medication increases with age, which can be attributed to the increasing prevalence of chronic diseases over the course of age [14, 79]. The European average for the use of prescribed medicines has remained similar compared to EHIS 2 (2014/2015) [80].

More than a third (37.3 %) of adults in Germany had taken non-prescribed medication in the two weeks prior to the survey. This prevalence is lower than in EHIS 2 (42.1 %) [9]. Significant gender-related differences in the use of non-prescribed medication were observed in both EHIS 2 and EHIS 3, with higher use prevalence among women than among men. Compared to Germany, the use of non-prescribed medication was lower than the European average at 33.3 %, but there were also gender differences, with significantly higher prevalence of use among women of all age groups.

The educational gradient both in Germany and in the European average runs in opposite directions for the use of prescribed and non-prescribed medication. People in the higher education group tend to take non-prescribed medication more often, while people in the lower education group take prescribed medication more often. This could indicate financial barriers. People with lower levels of education and income may have difficulty purchasing over-the-counter (OTC) medications and are more reliant on prescribed medications. In contrast, more educated people with higher income are more likely to be able to afford non-prescribed medications [81–83].

### 4.8 Strengths and weaknesses

The EHIS makes it possible to compare national health data with that of other European countries. The aim of the EHIS is to measure, on a harmonised basis and with a high degree of comparability between Member States, the state of health, the determinants of health, the utilisation of medical services and possible access barriers. This also allows for the analysis of socioeconomic inequalities in health and how these vary across Europe. The EHIS is therefore an important information basis for European health policy and reporting. Another strength is that all countries must follow detailed rules and recommendations for data collection to ensure a high degree of comparability [17, 80].

However, it should be noted that survey modes and sampling designs vary between countries, which must be considered when interpreting the results [81]. Furthermore, the data from EHIS are self-reported, which could have led to reporting and memory errors and thus to over- or underreporting. However, comparisons between countries should not be affected by this. When interpreting the results, it should also be noted that Germany is included in the calculations of the European average, so that the differences found between Germany and Europe tend to be conservative. It should be considered that the health systems of the countries and their financing vary. This includes, for example, the group of people entitled to benefits and the range of services that are offered or for which costs are covered. However, the comparative analyses at hand provide important insights. Even if the generalisability of the results is limited for the reasons mentioned, the European comparison based on EHIS 3 proves to be informative and meaningful.

### 4.9 Conclusion

What is particularly striking is the higher utilisation of all medical services considered in Germany compared to other European countries. However, a differentiated assessment must be made. For example, a comparison of utilisation does not readily allow statements to be made about the appropriateness or quality of care. Overall, high utilisation indicates good access to the healthcare system and an extensive range of services in Germany, but it can also be associated with overuse in some cases. If evidence-based measures such as colonoscopies or preventive services such as dental check-ups are used more frequently in Germany than in other countries, this can certainly be seen as positive. It seems that access to these services in Germany is organised with a low threshold and that the target groups are reached comparatively well.

The high level of inpatient service use in Germany is discussed more critically. A very high density of hospital beds by international comparison can result in increased utilisation and higher costs. However, financial incentives inherent in the system can also contribute to the high number of inpatient cases.

The criticism of the German healthcare system that a higher service density in Germany does not necessarily have a positive effect on the health of the population [55] points beyond the healthcare system. For example, life expectancy in Germany is developing less favourably than in other European countries. This is attributed not least to higher mortality from cardiovascular diseases [86], which is increasingly shifting the focus to the prevention of classic risk factors such as smoking, lack of exercise and alcohol consumption [55].

## Figures and Tables

**Table 1:** Case numbers for the indicators of the utilisation of medical services in Germany and Europe. Source: EHIS wave 3 (2018 – 2020)

	Women	Men	Total
**G P services**			
Germany	10,140	8,662	18,802
Europe	123,490	93,591	217,081
**Specialist services**			
Germany	8,634	6,328	14,962
Europe	87,938	59,312	147,250
**Dental services**			
Germany	10,619	8,873	19,492
Europe	92,932	71,539	164,471
**Psychiatric/psychotherapeutic services**			
Germany	1,341	788	2,129
Europe	10,268	5,670	15,938
**Hospital (inpatient)**			
Germany	2,048	1,867	3,915
Europe	16,021	13,130	29,151
**Colonoscopy in the past 10 years**			
Germany	3,467	3,019	6,486
Europe	18,509	16,541	35,050
**Cholesterol measurement in the past 12 months**			
Germany	7,844	6,720	14,564
Europe	91,870	72,405	164,275
**Blood sugar measurement in the past 12 months**			
Germany	7,499	6,469	13,968
Europe	93,043	72,362	165,405
**Blood pressure measurement in the past 12 months**			
Germany	9,794	7,885	17,679
Europe	110,097	85,004	195,101
**Medication: Not prescribed by a doctor**			
Germany	5,454	3,453	8,907
Europe	65,372	39,644	105,016
**Medication: Prescribed by a doctor**			
Germany	7,390	5,993	13,383
Europe	87,642	62,563	150,205

## Appendix

**Annex Table 1:** Age-standardised prevalence of utilisation of medical services by gender and age for Germany and Europe. Source: European Health Interview Survey (EHIS wave 3, 2018 – 2020)

	**General practitioner services in the past 12 months**				**Specialist services in the past 12 months**				**Psychiatric/psychotherapeutic services in the past 12 months**				**Hospital (inpatient) in the past 12 months**				**Blood pressure measurement in the past 12 months**				**Cholesterol measurement in the past 12 months**				**Blood sugar measurement in the past 12 months**				**Use of medication prescribed by a doctor in the last 2 weeks**				**Use of medication not prescribed by a doctor in the last 2 weeks**			
	**Germany**		**Europe**		**Germany**		**Europe**		**Germany**		**Europe**		**Germany**		**Europe**		**Germany**		**Europe**		**Germany**		**Europe**		**Germany**		**Europe**		**Germany**		**Europe**		**Germany**		**Europe**	
	**%**	**(95 % CI)**	**%**	**(95 % CI)**	**%**	**(95 % CI)**	**%**	**(95 % CI)**	**%**	**(95 % CI)**	**%**	**(95 % CI)**	**%**	**(95 % CI)**	**%**	**(95 % CI)**	**%**	**(95 % CI)**	**%**	**(95 % CI)**	**%**	**(95 % CI)**	**%**	**(95 % CI)**	**%**	**(95 % CI)**	**%**	**(95 % CI)**	**%**	**(95 % CI)**	**%**	**(95 % CI)**	**%**	**(95 % CI)**	**%**	**(95 % CI)**
**Women**	**83.6**	**(82.5 – 84.7)**	**79.3**	**(79.0 – 79.7)**	**67.8**	**(66.4 – 69.2)**	**58.4**	**(58.0 – 58.8)**	**13.5**	**(12.4 – 14.6)**	**7.7**	**(7.5 – 8.0)**	**17.0**	**(15.9 – 18.1)**	**10.9**	**(10.6 – 11.2)**	**80.1**	**(78.9 – 81.2)**	**69.4**	**(69.0 – 69.8)**	**62.7**	**(61.2 – 64.1)**	**57.5**	**(57.1 – 57.9)**	**60.0**	**(58.6 – 61.5)**	**57.8**	**(57.3 – 58.2)**	**56.2**	**(54.8 – 57.6)**	**51.2**	**(50.8 – 51.7)**	**43.5**	**(42.1 – 44.9)**	**38.6**	**(38.1 – 39.0)**
18 – 29 years	79.2	(75.6 – 82.4)	72.0	(70.9 – 73.1)	62.8	(58.5 – 66.9)	50.6	(49.3 – 52.0)	20.0	(16.5 – 23.9)	10.1	(9.2 – 11.2)	13.6	(10.7 – 17.0)	7.9	(7.1 – 8.9)	73.2	(69.2 – 76.7)	55.6	(54.3 – 56.9)	46.3	(41.9 – 50.7)	38.6	(37.3 – 39.9)	42.2	(37.8 – 46.7)	40.0	(38.7 – 41.3)	36.7	(32.6 – 40.9)	27.9	(26.7 – 29.2)	42.2	(38.1 – 46.3)	35.0	(33.8 – 36.3)
30 – 44 years	80.6	(78.0 – 83.0)	74.1	(73.4 – 74.9)	68.0	(64.9 – 71.0)	56.5	(55.6 – 57.4)	15.2	(13.0 – 17.6)	9.1	(8.5 – 9.7)	9.1	(5.7 – 14.2)	8.8	(8.2 – 9.5)	76.4	(73.6 – 78.9)	61.7	(60.8 – 62.6)	50.6	(47.4 – 53.8)	46.9	(46.0 – 47.8)	48.2	(45.0 – 51.5)	48.3	(47.3 – 49.2)	38.2	(35.2 – 41.3)	33.2	(32.3 – 34.1)	49.2	(46.0 – 52.3)	40.7	(39.8 – 41.6)
45 – 64 years	85.1	(83.5 – 86.6)	80.2	(79.7 – 80.7)	72.3	(70.3 – 74.2)	60.6	(59.9 – 61.2)	14.3	(12.8 – 15.9)	8.0	(7.6 – 8.4)	16.6	(15.1 – 18.3)	10.0	(9.6 – 10.5)	81.0	(79.2 – 82.7)	71.3	(70.8 – 71.9)	67.7	(65.7 – 69.7)	61.6	(60.9 – 62.2)	65.1	(63.0 – 67.2)	61.0	(60.4 – 61.7)	61.4	(59.3 – 63.4)	55.6	(54.9 – 56.2)	45.1	(43.0 – 47.2)	40.2	(39.6 – 40.9)
65 years and older	88.1	(86.5 – 89.5)	88.8	(88.4 – 89.3)	65.1	(62.7 – 67.4)	63.1	(62.3 – 63.8)	5.7	(4.7 – 6.9)	4.2	(3.9 – 4.5)	23.6	(21.6 – 25.7)	16.7	(16.1 – 17.3)	87.8	(86.2 – 89.3)	84.7	(84.2 – 85.2)	79.9	(78.0 – 81.6)	76.3	(75.7 – 76.9)	77.8	(75.8 – 79.7)	75.8	(75.2 – 76.4)	82.6	(80.9 – 84.2)	81.3	(80.8 – 81.8)	36.4	(34.2 – 38.7)	36.4	(35.8 – 37.1)
**Men**	**79.1**	(77.9 – 80.3)	**72.1**	**(71.7 – 72.5)**	**53.0**	**(51.6 – 54.5)**	**45.7**	**(45.3 – 46.2)**	**8.7**	(7.9 – 9.6)	**5.1**	**(4.8 – 5.3)**	**16.8**	**(15.7 – 17.9)**	**10.8**	**(10.4 – 11.1)**	**70.1**	**(68.7 – 71.5)**	**64.0**	**(63.5 – 64.4)**	**58.8**	**(57.3 – 60.3)**	**54.6**	**(54.1 – 55.1)**	**56.9**	**(55.4 – 58.4)**	**54.4**	**(53.9 – 54.8)**	**50.0**	**(48.5 – 51.4)**	**44.4**	**(43.9 – 44.8)**	**31.1**	**(29.8 – 32.5)**	**28.0**	**(27.6 – 28.4)**
18 – 29 years	76.2	(72.8 – 79.2)	62.1	(60.9 – 63.2)	42.5	(38.9 – 46.3)	33.3	(32.1 – 34.5)	8.9	(6.9 – 11.5)	5.9	(5.3 – 6.6)	9.3	(7.3 – 11.8)	5.4	(4.8 – 6.1)	50.5	(46.7 – 54.3)	43.3	(42.1 – 44.5)	33.4	(29.8 – 37.1)	30.6	(29.4 – 31.8)	32.0	(28.4 – 35.8)	31.1	(30.0 – 32.3)	21.0	(18.1 – 24.3)	16.5	(15.5 – 17.5)	36.3	(32.7 – 39.9)	27.0	(25.9 – 28.1)
30 – 44 years	71.8	(68.7 – 74.7)	63.1	(62.2 – 64.0)	43.8	(40.6 – 47.0)	35.4	(34.5 – 36.4)	9.5	(7.8 – 11.7)	5.9	(5.4 – 6.4)	10.5	(8.6 – 12.8)	6.0	(5.5 – 6.6)	59.6	(56.4 – 62.8)	52.5	(51.5 – 53.5)	44.6	(41.3 – 48.0)	41.6	(40.6 – 42.5)	42.5	(39.2 – 45.9)	41.5	(40.5 – 42.4)	31.3	(28.3 – 34.5)	24.5	(23.6 – 25.4)	36.1	(33.1 – 39.2)	30.0	(29.2 – 31.0)
45 – 64 years	79.9	(78.0 – 81.8)	73.4	(72.8 – 74.0)	55.7	(53.3 – 58.1)	47.5	(46.8 – 48.3)	11.5	(9.9 – 13.3)	5.9	(5.4 – 6.3)	19.0	(17.1 – 21.0)	11.2	(10.6 – 11.7)	76.7	(74.6 – 78.6)	68.6	(67.9 – 69.3)	66.5	(64.1 – 68.7)	60.2	(59.5 – 60.9)	64.2	(61.8 – 66.5)	59.4	(58.7 – 60.1)	56.2	(53.8 – 58.6)	48.8	(48.1 – 49.5)	28.2	(26.2 – 30.4)	27.5	(26.8 – 28.1)
65 years and older	88.0	(86.0 – 89.8)	86.8	(86.2 – 87.3)	67.0	(64.4 – 69.5)	62.8	(62.0 – 63.6)	3.8	(3.0 – 4.8)	2.5	(2.3 – 2.8)	26.0	(23.6 – 28.4)	18.9	(18.2 – 19.6)	86.7	(84.6 – 88.5)	83.9	(83.3 – 84.5)	80.5	(78.1 – 82.7)	76.8	(76.1 – 77.5)	79.0	(76.5 – 81.2)	76.6	(75.9 – 77.3)	82.9	(80.7 – 84.9)	78.8	(78.1 – 79.5)	26.0	(23.8 – 28.3)	27.3	(26.6 – 28.0)
**Total**	**81.4**	(80.5 – 82.2)	**75.7**	(75.5 – 76.0)	**60.4**	**(59.4 – 61.4)**	**52.1**	**(51.8 – 52.5)**	**11.1**	**(10.4 – 11.8)**	**6.4**	**(6.2 – 6.6)**	**16.9**	**(16.1 – 17.7)**	**10.8**	**(10.6 – 11.1)**	**75.1**	**(74.2 – 76.0)**	**66.7**	**(66.4 – 67.0)**	**60.7**	**(59.7 – 61.7)**	**56.1**	**(55.7 – 56.4)**	**58.5**	**(57.4 – 59.5)**	**56.1**	**(55.8 – 56.4)**	**53.1**	**(52.1 – 54.1)**	**47.8**	**(47.5 – 48.2)**	**37.3**	**(36.3 – 38.3)**	**33.3**	**(33.0 – 33.6)**
18 – 29 years	77.7	(75.3 – 79.9)	67.1	(66.3 – 67.9)	52.6	(49.8 – 55.4)	42.0	(41.1 – 42.9)	14.4	(12.3 – 16.8)	8.0	(7.4 – 8.7)	11.4	(9.6 – 13.5)	6.7	(6.1 – 7.2)	61.8	(59.0 – 64.5)	49.5	(48.6 – 50.4)	39.8	(37.0 – 42.8)	34.6	(33.7 – 35.5)	37.0	(34.2 – 40.0)	35.6	(34.7 – 36.5)	28.8	(26.2 – 31.5)	22.2	(21.4 – 23.1)	39.2	(36.5 – 42.0)	31.0	(30.2 – 31.9)
30 – 44 years	76.2	(74.2 – 78.1)	68.7	(68.1 – 69.3)	55.9	(53.6 – 58.2)	46.1	(45.4 – 46.8)	12.4	(10.9 – 13.9)	7.5	(7.1 – 7.9)	12.1	(10.6 – 13.8)	7.4	(7.0 – 7.9)	68.0	(65.9 – 70.1)	57.2	(56.5 – 57.8)	47.7	(45.4 – 50.0)	44.3	(43.6 – 45.0)	45.4	(43.1 – 47.7)	44.9	(44.3 – 45.6)	34.8	(32.6 – 37.0)	28.9	(28.2 – 29.5)	42.6	(40.4 – 44.9)	35.4	(34.8 – 36.1)
45 – 64 years	82.5	(81.2 – 83.7)	76.9	(76.4 – 77.3)	64.0	(62.4 – 65.6)	54.1	(53.6 – 54.6)	12.9	(11.8 – 14.1)	6.9	(6.6 – 7.2)	17.8	(16.6 – 19.1)	10.6	(10.2 – 11.0)	78.8	(77.5 – 80.1)	70.0	(69.5 – 70.4)	67.1	(65.6 – 68.6)	60.9	(60.4 – 61.4)	64.7	(63.1 – 66.2)	60.2	(59.7 – 60.7)	58.8	(57.2 – 60.4)	52.2	(51.7 – 52.7)	36.7	(35.2 – 38.2)	33.9	(33.5 – 34.4)
65 years and older	88.1	(86.8 – 89.2)	87.8	(87.4 – 88.2)	66.1	(64.3 – 67.8)	62.9	(62.4 – 63.5)	4.8	(4.1 – 5.5)	3.4	(3.2 – 3.6)	24.8	(23.2 – 26.4)	17.8	(17.3 – 18.3)	87.3	(86.0 – 88.5)	84.3	(83.9 – 84.7)	80.2	(78.7 – 81.6)	76.6	(76.1 – 77.0)	78.4	(76.8 – 79.9)	76.2	(75.7 – 76.7)	82.8	(81.4 – 84.0)	80.1	(79.6 – 80.5)	31.2	(29.6 – 32.8)	31.9	(31.4 – 32.4)

% = percent; 95 % CI = 95 % confidence interval

**Annex Table 2:** Age-standardised prevalence of utilisation of dental services by gender and age for Germany and Europe. Source: European Health Interview Survey (EHIS wave 3, 2018 – 2020)

	Dental services in the past 12 months			
	Germany		Europe	
	%	(95 % CI)	%	(95 % CI)
**Women**	**86.1**	**(84.9 – 87.1)**	**64.6**	**(64.2 – 65.0)**
18 – 29 years	82.6	(78.9 – 85.7)	68.0	(66.8 – 69.2)
30 – 44 years	90.2	(87.9 – 92.1)	69.6	(68.9 – 70.4)
45 – 64 years	88.9	(87.3 – 90.4)	67.1	(66.5 – 67.7)
65 – 74 years	85.2	(82.5 – 87.6)	59.2	(58.3 – 60.2)
75 years and older	74.8	(71.3 – 78.0)	45.4	(44.3 – 46.5)
**Men**	**78.6**	**(77.3 – 79.9)**	**57.6**	**(57.2 – 58.1)**
18 – 29 years	76.2	(72.7 – 79.4)	59.8	(58.6 – 61.0)
30 – 44 years	78.0	(74.9 – 80.7)	60.4	(59.5 – 61.4)
45 – 64 years	79.6	(77.4 – 81.6)	59.0	(58.3 – 59.7)
65 – 74 years	79.7	(76.3 – 82.8)	54.6	(53.5 – 55.6)
75 years and older	79.9	(76.5 – 82.9)	47.3	(46.1 – 48.6)
**Total**	**82.3**	**(81.5 – 83.2)**	**61.1**	**(60.8 – 61.5)**
18 – 29 years	79.4	(76.9 – 81.6)	63.9	(63.1 – 64.8)
30 – 44 years	84.1	(82.2 – 85.8)	65.1	(64.5 – 65.7)
45 – 64 years	84.2	(82.9 – 85.5)	63.1	(62.6 – 63.6)
65 – 74 years	82.5	(80.3 – 84.5)	56.9	(56.2 – 57.6)
75 years and older	77.4	(74.9 – 79.6)	46.4	(45.5 – 47.2)

% = percent; 95 % CI = 95 % confidence interval

**Annex Table 3:** Age-standardised prevalence of utilisation of colonoscopy by gender and age for Germany and Europe. Source: European Health Interview Survey (EHIS wave 3, 2018 – 2020)

	Colonoscopy in the past 10 years			
	Germany		Europe	
	%	(95 % CI)	%	(95 % CI)
**Women**	**53.6**	**(51.7 – 55.4)**	**28.9**	**(28.3 – 29.5)**
50 – 54 years	38.6	(34.8 – 42.5)	20.9	(19.7 – 22.1)
55 – 59 years	49.1	(45.4 – 52.8)	25.8	(24.7 – 27.0)
60 – 64 years	58.7	(54.7 – 62.6)	31.0	(29.8 – 32.3)
65 – 69 years	60.1	(55.9 – 64.1)	33.4	(32.1 – 34.7)
70 – 74 years	67.1	(62.4 – 71.4)	36.6	(35.1 – 38.2)
**Men**	**51.6**	**(49.5 – 53.7)**	**29.8**	**(29.1 – 30.4)**
50 – 54 years	34.8	(30.6 – 39.2)	19.6	(18.3 – 20.8)
55 – 59 years	43.3	(39.0 – 47.7)	25.6	(24.3 – 26.9)
60 – 64 years	58.5	(54.1 – 62.7)	32.6	(31.3 – 34.0)
65 – 69 years	61.8	(57.3 – 66.0)	35.8	(34.5 – 37.2)
70 – 74 years	66.4	(60.6 – 71.8)	39.2	(37.5 – 40.9)
**Total**	**52.6**	**(51.2 – 54.0)**	**29.3**	**(28.9 – 29.8)**
50 – 54 years	36.7	(33.8 – 39.6)	20.2	(19.4 – 21.1)
55 – 59 years	46.2	(43.3 – 49.1)	25.7	(24.9 – 26.6)
60 – 64 years	58.6	(55.6 – 61.5)	31.8	(30.9 – 32.8)
65 – 69 years	60.9	(57.9 – 63.9)	34.6	(33.7 – 35.6)
70 – 74 years	66.8	(63.1 – 70.3)	37.9	(36.8 – 39.0)

% = percent; 95 % CI =95 % confidence interval
